# PKN2 Inhibits VEGFA and bFGF‐Mediated Angiogenesis by Targeting HIF‐1α in Colon Cancer

**DOI:** 10.1002/kjm2.70050

**Published:** 2025-06-14

**Authors:** Yun Zhu, Shi‐Yun Huang, Yi Lei, An‐Ni Luo, Xiang‐Zhao Li, Biao Wang, Peng‐Hui Sun, Si‐Tang Gong, Yang Cheng

**Affiliations:** ^1^ Department of Infectious Diseases Nanfang Hospital, Southern Medical University, State Key Laboratory of Organ Failure Research, Key Laboratory of Infectious Diseases Research in South China, Ministry of Education, Guangdong Provincial Key Laboratory for Prevention and Control of Major Liver Diseases, Guangdong Provincial Clinical Research Center for Viral Hepatitis, Guangdong Institute of Hepatology, Guangdong Provincial Research Center for Liver Fibrosis Engineering and Technology Guangzhou Guangdong China; ^2^ Digestive Department, Guangzhou Women and Children's Medical Center Guangzhou Medical University Guangzhou Guangdong China; ^3^ Department of Pathology, Nanfang Hospital Southern Medical University Guangzhou Guangdong China; ^4^ Department of Hepatobiliary Surgery, Nanfang Hospital Southern Medical University Guangzhou Guangdong China; ^5^ Nanfang PET Center, Nanfang Hospital Southern Medical University Guangzhou Guangdong China; ^6^ Digestive Department Liuzhou Hospital, Guangzhou Women and Children's Medical Center Guangzhou Medical University Liuzhou Guangxi China

**Keywords:** colon cancer, HIF‐1α, PKN2, tumor angiogenesis

## Abstract

Angiogenesis plays a vital role in colon cancer growth and metastasis. The role of protein kinase N2 (PKN2) in colon cancer is rarely studied. In this study, we investigated the effect of PKN2 on angiogenesis in colon cancer. We evaluated the correlation between PKN2 expression and microvessel density (MVD) in tumor tissue of patients with colon cancer. The effect of PKN2 on tumor angiogenesis was investigated both in cultured colon cancer cells and in a mouse colon cancer model. PKN2 targeted vascular endothelial growth factor A (VEGFA) and basic fibroblast growth factor (bFGF) expression, and secretion were analyzed, and the specific regulatory role of PKN2 on HIF was explored. PKN2 expression was negatively correlated with tumor MVD in tumor tissue of patients with colon cancer. PKN2 inhibited angiogenesis in both in vitro and in vivo models of mouse tumors. Mechanistically, PKN2 suppressed the transcriptional activity of hypoxia‐inducible factor‐1α (HIF‐1α) and reduced its nuclear accumulation, leading to the inhibiting of VEGFA and bFGF transcription by preventing HIF‐1α binding to their promoters. Additionally, PKN2 directly interacted with HIF‐1α at the protein level and induced phosphorylation, resulting in ubiquitination‐dependent degradation of HIF‐1α in colon cancer cells. Our study demonstrated, for the first time, that PKN2 exerts inhibitory effects on tumor angiogenesis in colon cancer. We propose a novel mechanism by which PKN2 regulates VEGFA and bFGF expression through modulation of the dynamic equilibrium of HIF‐1α protein levels.

## 1 Introduction

Colon cancer is the most prevalent malignancy worldwide, with the third‐highest incidence and the second‐highest mortality rate globally [1]. At initial diagnosis, approximately 20% of patients with primary colon cancer have metastatic disease. Moreover, tumor relapse and metastasis account for 30%–50% of deaths in patients who undergo curative surgery for colorectal cancer. Furthermore, the five‐year survival rate for metastatic colorectal cancer remains low (10%) [2].

Angiogenesis plays a pivotal role in the progression and metastasis of colon cancer, representing a complex process regulated by multiple proangiogenic factors. Among these factors, vascular endothelial growth factor (VEGF) and basic fibroblast growth factor (bFGF) are major potent angiogenic regulators [3]. Consequently, targeting VEGF and bFGF signaling pathways has become the primary focus of studies on developing anticancer agents [4]. Currently, some angiogenesis inhibitors that specifically target the VEGF signaling pathway have been approved for treating metastatic colorectal cancer. Additionally, clinical trials are underway to investigate inhibitors of FGF signaling as potential therapeutic options. However, these inhibitors may have significant side effects, such as bleeding and cardiovascular toxicity. Besides, their use may lead to hypoxia, which promotes VEGF production and subsequently contributes to tumor progression and drug resistance [5, 6]. Therefore, there is an urgent need to explore novel antiangiogenesis therapeutic strategies to treat colon cancer effectively.

Protein kinase N (PKN) belongs to the PKC‐related serine/threonine‐protein kinase family and has three isoforms (PKN1, PKN2, and PKN3). These isoforms are widely expressed in mammalian tissues, with varying expression levels [7]. Parker et al. first described PKN2 in 1994 [8]. It acts as an effector of Rho GTPases in various cellular pathways. PKN2 has been recognized for its critical roles in cell cycle regulation, cell adhesion, actin stress fiber formation, and apical junction maturation promotion across cells of different tissues. Additionally, it is involved in tumor cell migration, invasion, and apoptosis in several cancers, including bladder, prostate, and breast cancers [9]. However, the role of PKN2 in colon cancer remains poorly investigated. In our previous study [10], we observed a high expression of PKN2 in colon cancer cells that inhibited tumor growth by suppressing M2‐like polarization of tumor‐associated macrophages. This study aims to investigate the effects of PKN2 on colon cancer angiogenesis under hypoxic and normoxic conditions.

## 2 Materials and Methods

### 2.1 Human Samples and Immunohistochemistry

Ninety colon cancer samples were collected from patients who underwent surgery or biopsy at Nanfang Hospital between June 2007 and April 2010. We adhered to the declaration of Helsinki and obtained approval from the Ethics Committee of Nanfang Hospital before the commencement of the study. Paraffin‐embedded tumor tissue sections were stained using PKN2 (Abcam, ab314021) and CD31 antibodies (Abcam, ab182981), following previously described protocols [11]. To ensure specificity, a negative control was included by performing control staining with only a secondary antibody (Rabbit IgG polyclonal‐isotype, Abcam, ab37415) Two experienced pathologists independently assessed the PKN2 staining score. CD31 expression was quantified using ImageJ software by measuring the integrated optical density (IOD). Individual endothelial cells or clusters of endothelial cells that stained brown and were not associated with adjacent microvessels, tumor cells, or other tissues were considered single microvessels. The number of microvessels was determined using the HALO platform colocalization v1.3 algorithm.

### 2.2 Confocal Laser Scanning Microscopy

Tissue paraffin sections were sequentially dehydrated in xylene and gradient alcohol. Following antigen retrieval by heating, the sections were washed with PBS and immersed in 3% hydrogen peroxide. Subsequently, the sections were rinsed with PBS and incubated at 37°C for 15 min with 5% bovine serum albumin. Afterward, the sections were incubated overnight at 4°C with primary antibodies against PKN2 (Abcam, ab238334) and CD31. The next day, Alexa‐488‐ or Alexa‐594‐conjugated secondary antibodies (Abcam, ab150081& ab150084) were applied to the sections for 30 min. Finally, confocal laser scanning microscopy was used to view the slides after mounting them in an antifade reagent containing DAPI (Thermo Fisher, 62248).

### 2.3 Cell Culture and Transfection

Human colon cancer cell lines (HCT116, HCT8, HT29, and SW480) and the human umbilical vein cell fusion cell EA.hy926 were obtained from the Cell Bank of the Typical Culture Collection Committee at the Chinese Academy of Sciences. HCT116 and HCT8 cells were cultured in RPMI 1640 medium (Gibco, 11875168) supplemented with 10% fetal bovine serum (FBS). HT29 cells were cultured in DMEM (Gibco, 11320033) supplemented with 10% FBS, and EA.hy926 cells were cultured in an endothelial cell growth medium (ECGM; PromoCell, C‐22010). The colon cancer cell lines were transfected with plasmids and shRNA using the method described in our previous study [10]. The PKN2‐K686R plasmid was generated by introducing a K686R point mutation at the ATP binding site of the PKN2‐WT plasmid. Stable cell lines were selected. Regular cell culture was performed in an incubator with a gas concentration of 5% CO_2_, 21% O_2_, and 74% N_2_. Anaerobic culture was conducted by adjusting the gas concentrations to 0.5% O_2_, 5% CO_2_, and 94.5% N_2_.

### 2.4 Wound‐Healing Assay

Ten percent of the culture supernatant of the treated colon cancer cells was added to the EA.hy926 cell culture medium. The EA.hy926 cells were seeded onto 24‐well plates and cultured until they reached confluence. After 12 h of serum starvation, the cells were gently wounded using a pipette tip. Subsequently, the injured cells were immediately treated. Images were captured immediately after wounding and again after 24 h to evaluate cell motility by measuring the distance between the wound edges.

### 2.5 Tube Formation Assays

IbiTreat slides (Ibidi, 83612) were used for the experiment. The lower wells were inoculated with liquid Matrigel (Corning, 354230) and allowed to solidify at 37°C for 1 h. Subsequently, EA.hy926 cells were seeded onto the surface of Matrigel and cultured in serum‐free ECGM supplemented with 10% culture supernatant from treated colon cancer cells. The EA.hy926 cells were then incubated for 12 h. After this, nine randomly selected fields were photographed, and the enclosed networks of fully formed tubes within these fields were quantified.

### 2.6 Transcription Factor Activity Array

The nuclear extracts from HT29 cells treated using the Nuclear Extract Kit (Thermo Fisher, 78835) were prepared according to the manufacturer's instructions. Subsequently, transcription factor array (Thermo Fisher, 4418784) analysis was performed as described in our previous study [10]. A total of 345 transcription factors were detected, and spots exhibiting ≥ 2‐fold change were considered statistically significant.

### 2.7 Western Blot and Immunoprecipitation

Total protein and nuclear protein were extracted from cultured cells, and a Western blot analysis was performed as previously described [12]. Primary antibodies targeting PKN2 (Abcam, ab314021), hypoxia‐inducible factor‐1α (HIF‐1α; Abcam, ab51608), pHIF‐1α (AmyJet Scientific, pab11030), and flag (Cell Signaling, 14793) were used. Pretreated cells were washed with precooled PBS and then lysed with precooled lysis buffer on ice for 30 min. Subsequently, the cell lysates were centrifuged at 12,000 × *g* for 10 min to obtain supernatants. The supernatants were incubated overnight at 4°C on a rotator with anti‐PKN2 (Abcam, ab87812) or control IgG, followed by the addition of 30 μL of prewashed protein A/G agarose beads (Proteintech, PR40025) for 2 h. After thorough washing using diluted lysis buffer, the beads were used for Western blot analysis.

### 2.8 RT‐PCR

Total RNA was extracted from colon cancer cell lines treated with Trizol reagent (Thermo Fisher, 15596018CN) following the manufacturer's instructions. The mRNA was reverse transcribed from 500 ng of total RNA using the Prime Script RT reagent kit (Takara Bio Inc., RR037A) according to the manufacturer's protocol. Per the protocol, quantitative real‐time RT‐PCR of target genes was performed using specific primers and Thermal Cycler Dice Real Time (Takara Bio Inc., TP807). Data were normalized to GAPDH, a housekeeping gene, and analyzed using StepOne software with the ∆∆CT method. Table S1 lists the primers used.

### 2.9 Chromatin Immunoprecipitation Assay (ChIP)

ChIP analysis was performed on pretreated colon cancer cells using the Pierce Magnetic ChIP Kit (Thermo Fisher, 26157) according to the manufacturer's instructions. Briefly, cells were cross‐linked with 1% formaldehyde for 10 min at room temperature and then washed twice with ice‐cold PBS containing protease inhibitors. The cell pellets were washed with PBS and resuspended in ChIP lysis buffer on ice for 10 min. Following sonication, the lysates were diluted with ChIP dilution buffer and protein A beads were added to the lysates; the mixture was then rotated at 4°C for 1 h. The supernatants were incubated with antibodies against HIF‐1α or without antibodies at 4°C for 2 h. Immune complexes were recovered by adding blocked protein A beads and incubating them overnight at 4°C. Beads underwent sequential washing, followed by elution using an elution buffer. The mixture was then incubated at 65°C for 4 h to reverse the cross‐links formed during earlier steps of the procedure. After digestion with proteinase K, DNA fragments were extracted before being resuspended and used in RT‐PCR reactions.

### 2.10 Ubiquitination Assay

Before harvesting, the cells were treated with 5 μM MG132 (Selleck Chemicals, S6219) for 6 h and washed with PBS. The cells were then lysed using IP lysis/wash buffer containing protease and phosphatase inhibitors(Thermo Fisher, 78443), as well as 10 μM N‐ethylmaleimide (NEM; Sigma, E3876), on ice for 30 min. After quantification of cleared lysates, immunoprecipitation with protein A/G agarose (Sigma, IP10) prebound to specified antibodies was performed on equal amounts of each lysate. The resin beads were washed with lysis buffer before elution and separation on an SDS‐PAGE gel. Immunoblotting was performed using the indicated antibodies.

### 2.11 Animals and Xenograft Models

The Animal Care Committee of the Southern Medical University approved the animal protocols to ensure compliance with ethical standards. Female BALB/c nude mice, aged 4–6 weeks, were housed in a pathogen‐free environment throughout the experiment. Mice (*n* = 5) were subcutaneously injected with HT‐29 cells stably transfected with the control vector or PKN2 (2.5 × 10^6^/mice). The tumor size was measured every other day using a vernier caliper, and the tumor volume was calculated using the formula: Volume = $\frac{\text{length}\times {\text{width}}^{2}}{2}$.

After 15 days postinjection, the mice were euthanized, and their tumors were collected for subsequent confocal assay and enzyme‐linked immunosorbent assay (ELISA). The same treatment was administered to the other two groups of mice (*n* = 10) to perform a survival analysis and body weight measurement.

### 2.12 ELISA

Mice subcutaneous tumors were excised and rinsed to remove the blood. Each tumor (100 mg) was homogenized and sonicated for 10 min. The homogenates were then centrifuged at 12,000 × *g* for 10 min. Following the provided instructions, VEGFA and bFGF levels in the supernatants of the homogenates or cell culture were quantified using ELISA (Abcam, ab119565) (Thermo Fisher, EMFGF2).

### 2.13 Luciferase Assay

The luciferase reporter plasmids were constructed by ligating −1000 to 0 bp of VEGFA and bFGF promoter sequences to the pGL3 control plasmid (Promega, E1741). PKN2 stably transfected HCT118 cells, and control cells were plated at a 1.5 × 10^5^ cells/well density in 24‐well plates. Cells were then transfected with a luciferase reporter plasmid (200 ng) in combination with 80 ng pRL‐TK (Promega, E2241) using Lipofectamine 2000 (Invitrogen, 11668019). After 48 h of transfection, the medium was removed, and cells were rinsed twice with PBS buffer. Cells were lysed, followed by centrifugation at 12,000 rpm for 5 s. Luciferase activity was measured using the Dual‐Luciferase Reporter assay system (Promega, E1910). Firefly luciferase activity was normalized to Renilla luciferase activity.

### 2.14 Statistical Analysis

The data from at least three experiments were presented as means ± SEM. Student *t*‐tests were used to compare continuous variables between the control and treatment groups. The Kaplan–Meier method was used for the overall survival analysis, and a log‐rank test was used to compare differences between groups. A Spearman's correlation analysis was conducted to assess the correlation between PKN2 expression and both the IOD of CD31 and microvessel density (MVD). The sample size for Spearman's rank correlation analysis was determined using the PASS software based on an effect size of 0.5, a power of 0.9, and an *α* of 0.05. The estimated sample size was 53. All statistical analyses were performed using SPSS 13.0 (SPSS Inc.). Two‐way ANOVA was used to compare mice's tumor volume and body weight between groups. A *p* of < 0.05 was considered statistically significant.

## 3 Results

### 3.1 PKN2 Expression Correlated With Tumor Microvessel Density in Patients With Colon Cancer

**FIGURE 1:**
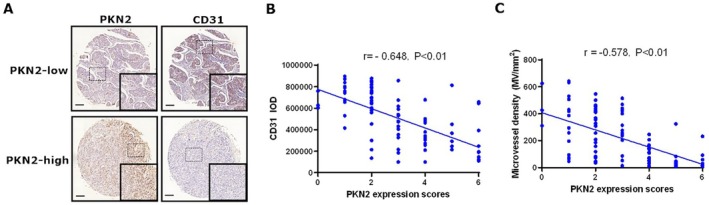
PKN2 expression correlated with MVD and CD31 in colon cancer tissue. (A) IHC staining of CD31 and PKN2 in colon cancer tissue from patients with low versus high PKN2 expression (*n* = 45); typical pictures are shown (100× and 400×). (B) Correlation between the PKN2 expression score and MVD using the Spearman method. (C) Correlation between the PKN2 expression score and the IOD of CD31 using the Spearman method.

The correlation between PKN2 expression with both CD31 expression and MVD was investigated in a cohort of 90 colon cancer tissue samples. Microvessel formation in colon cancer was assessed by immunohistochemical (IHC) staining for CD31 expression (Figure 1A) and MVD. Patients were divided into PKN2 high and low expression groups according to IHC staining (*n* = 45). Patients with high PKN2 expression showed significantly lower CD31 staining intensity compared to those with low PKN2 expression. As depicted in Figure 1B, there was an inverse association between PKN2 and CD31 expression in colon cancer tissues. Furthermore, there was a negative association between PKN2 expression and MVD (Figure 1C). These findings suggest that PKN2 expression negatively correlates with angiogenesis in tumor tissues.

### 3.2 PKN2 Inhibits Angiogenesis Induced by Colon Cancer Cells

**FIGURE 2:**
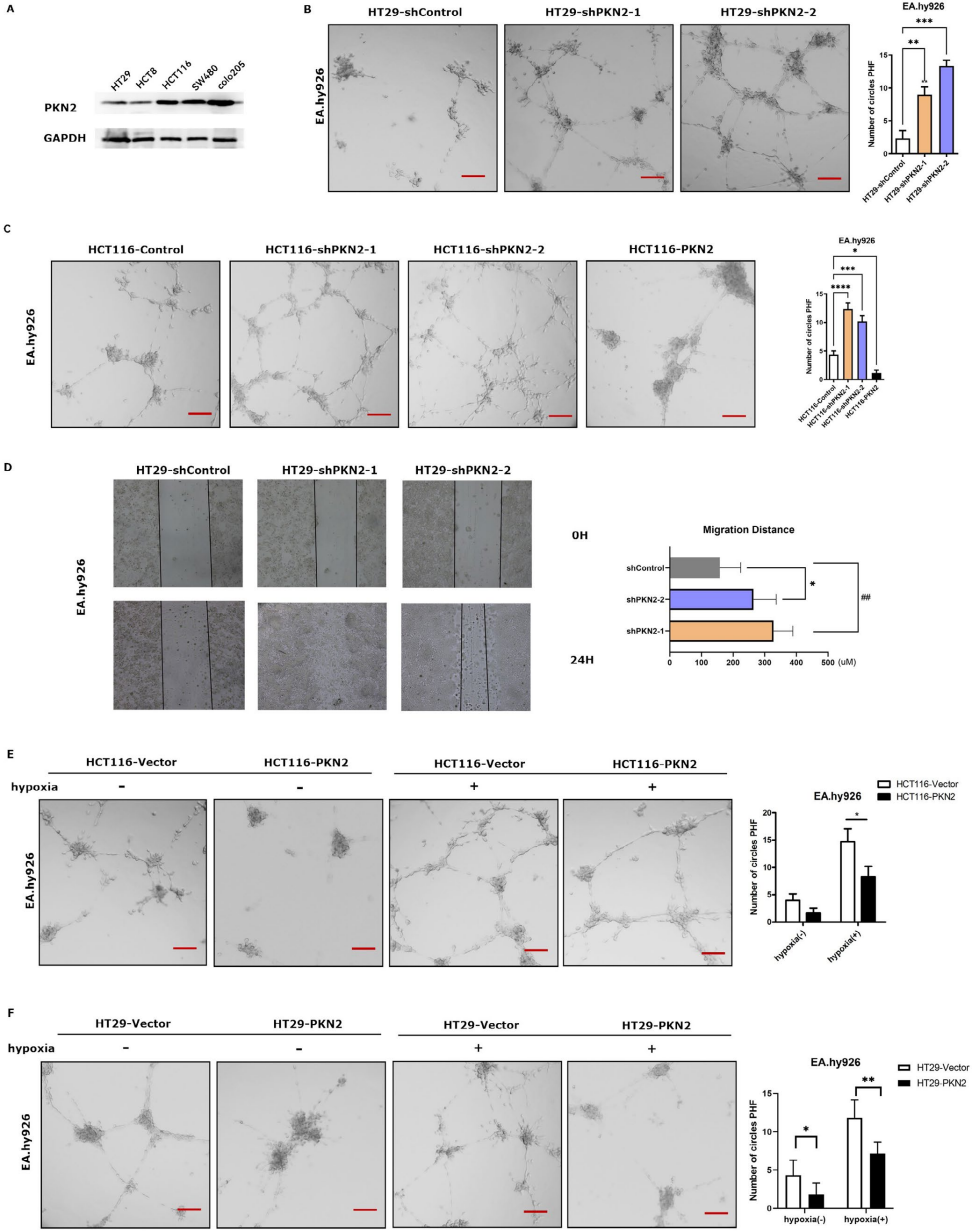
Effect of PKN2 on the proangiogenic effect of colon cancer cells in vitro. (A) The expression of PKN2 in various colon cancer cells. (B) Tube formation by EA.hy926 cells treated with conditioned medium from control shRNA or shPKN2 HT29 cells was assessed. (C) Tube formation by EA.hy926 cells treated with conditioned medium from control, PKN2 knockdown, or PKN2 overexpression HCT116 cells was assessed. (D) EA.hy926 cells were treated as indicated in section B and a migration assay was conducted. Quantification was conducted by measuring the migrated distance. (E) HCT116 or HT29 cells (F) stably overexpressing PKN2 or a vector cultured aerobically and anaerobically for 12 h. The supernatant was used to treat EA.hy926 cells, and tube formation was subsequently evaluated (200×). *p < 0.05, **p < 0.01, ***p < 0.001.

The expression of PKN2 was examined across various human colon cancer cell lines (Figure 2A). We established an in vitro tumor microvessel model using a coculture system. The conditioned medium from colon cancer cells was used on EA.hy926 cells in angiogenesis experiments. As depicted in Figure 2B,C, the supernatant from HT29 cells and HCT116 cells with stable PKN2 knockdown significantly enhanced tube formation compared to control cells. Conversely, the supernatant from HCT116 cells overexpressing PKN2 markedly inhibited tube formation compared to the control (Figure 2C). Additionally, the culture supernatants from shPKN2‐HT29 and shControl‐HT29 cells were employed as conditioned media for EA.hy926 cell migration assays. The results demonstrated that the culture supernatant from shPKN2‐HT29 cells more effectively promoted the migration of EA.hy926 cells than that from shControl‐HT29 cells (Figure 2D). These findings suggest that PKN2 in colon cancer cells inhibits the migration of EA.hy926 cells. HCT116 cell lines that stably expressed either PKN2 or the control vector were cultured under normoxic or hypoxic conditions. Figure 2C illustrates an increase in tube formation by EA.hy926 when cultured with HCT116 or HT29 cell supernatant under hypoxic conditions (Figure 2E,F; lane one vs. three, lane two vs. four). Notably, overexpressed PKN2 from colon cancer cells significantly reduced tube formation by EA.hy926 cells under both normoxic and hypoxic conditions (Figure 2E,F; lane one vs. three, lane two vs. four). These results indicate that PKN2 can inhibit hypoxia‐induced tube formation by colon cancer cells.

### 3.3 PKN2 Inhibited the Expression of VEGFA and bFGF in Colon Cancer Cells

**FIGURE 3:**
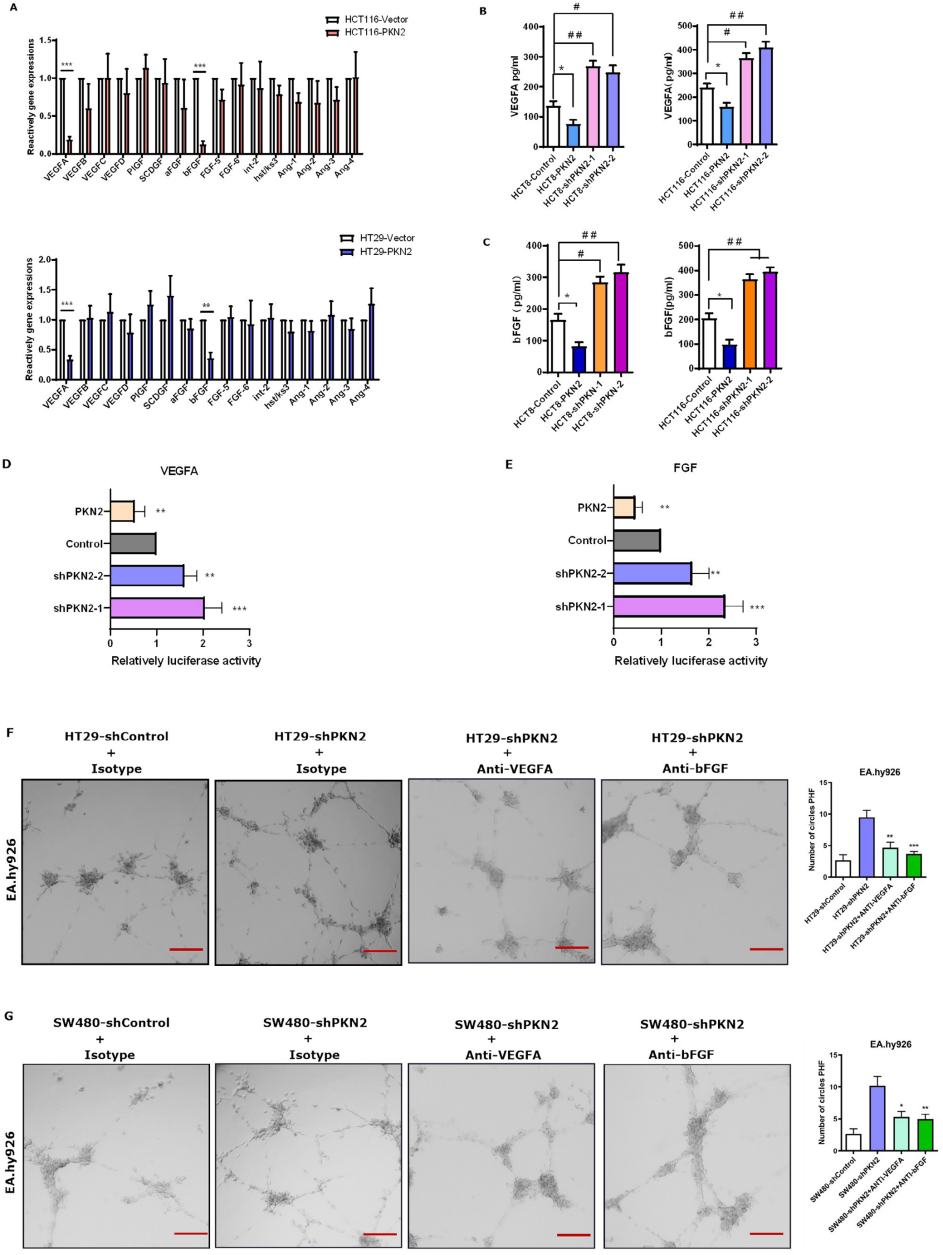
PKN2 suppressed the expression and secretion of VEGFA and bFGF in colon cancer cells, leading to impaired angiogenesis. (A) RT‐qPCR of proangiogenesis factors in HCT116 and HT29 cells with a PKN2 vector. (B, C) Levels of VEGFA and bFGF in the supernatants from control, PKN2 overexpression, and PKN2 knockdown HCT8 cells and HCT116 cells. (D) and (E) HCT116 cells were stably infected as indicated. The transcription factor binding activities of VEGFA and bFGF (E) were detected. Relative fold‐change in luciferase activity is shown. (F) and (G) Conditioned medium from PKN2 knockdown or control HT‐29 and SW480 (G) cells with human VEGFA, bFGF antibodies, or control isotype was used for the tube formation assay for EA.hy926 cells (200×). *p < 0.05, **p < 0.01, ***p < 0.001.

We hypothesized that PKN2 modulates angiogenesis by influencing the paracrine effect of colon cancer cells. RT‐qPCR was performed to assess the expression levels of proangiogenic factors by cells with PKN2 overexpression and control colon cancer cells. Our findings demonstrated a significant reduction in VEGFA and bFGF expression in PKN2‐overexpressing colon cancer cells compared to control cells, while other proangiogenic factors remained unchanged (Figure 3A). Additionally, we investigated the levels of secreted VEGFA and bFGF in the culture supernatant of PKN2‐overexpressing, PKN2‐knockdown, and control colon cancer cells. A significant decrease in VEGFA and bFGF secretion was observed in PKN2‐overexpressing cells compared to that in control cells, while VEGFA and bFGF levels were elevated in PKN2‐knockdown HCT8, HCT116 cells (Figure 3B) as well as SW480 cells (Figure S1A). Furthermore, luciferase reporter assays revealed significantly decreased promoter activities for both VEGFA and bFGF genes in PKN2‐overexpressing HCT116 cells, whereas increased activities were observed in PKN2‐knockdown cells (Figure 3D,E). Similar results were obtained in SW480 cells (Figure S1B). These findings indicate that PKN2 can inhibit both the expression and secretion of VEGFA and bFGF in colon cancer cells.

Additionally, human anti‐VEGFA, human anti‐bFGF, and isotype control antibodies were introduced into the conditioned culture supernatant of EA.hy926 cells, as depicted in Figure 2C. As illustrated in Figure 3F,G, the supernatant from PKN2 knockdown colon cancer cells significantly enhanced tube formation by EA.hy926 cells. At the same time, both VEGFA and bFGF antibodies attenuated the proangiogenic potential of the conditioned medium derived from PKN2‐knockdown cells. These findings suggest that PKN2 suppresses the proangiogenic effect of colon cancer cells by inhibiting the expression and secretion of VEGFA and bFGF.

### 3.4 PKN2 Inhibited Tumor Angiogenesis in a Mouse Colon Cancer Model

**FIGURE 4:**
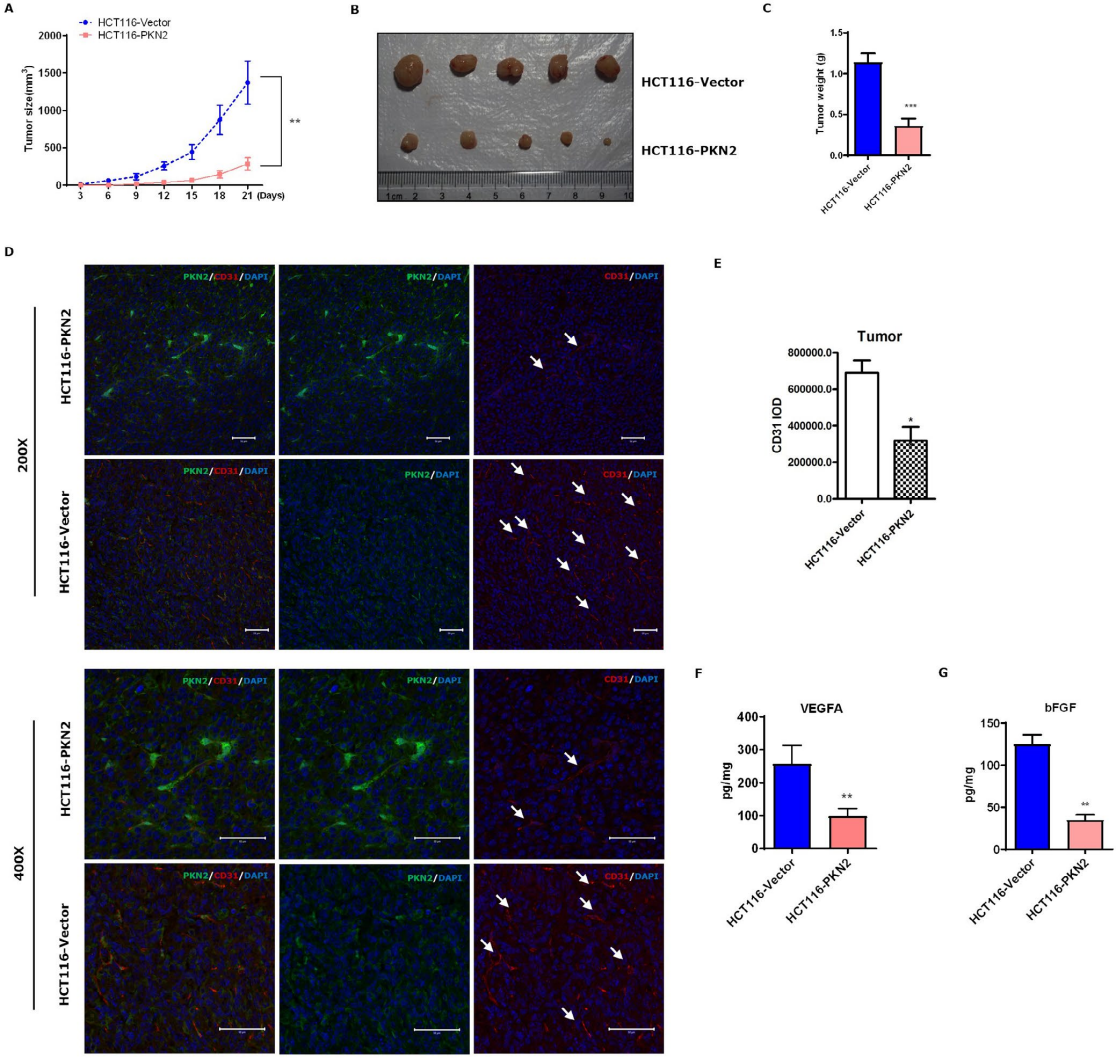
Effect of PKN2 on colon cancer growth and angiogenesis in mice. (A) The tumor diameter in nude mice following subcutaneously injection of overexpressed PKN2 and control HCT116 cells. (B) Representative photos of the subcutaneous tumors. (C) The tumor weight in both groups of nude mice. (D) Confocal assay of CD31 (red) and PKN2 (green) in tumor tissues (200× and 400×). The arrows indicate microvessels. (E) The IOD of CD31 staining. (F) and (G) VEGFA and bFGF levels in tumor tissue samples were determined by ELISA. *p < 0.05, **p < 0.01, ***p < 0.001.

The subcutaneous tumor model in BALB/c nude mice was established using HCT116 cells that stably expressed PKN2 or the control vector. Overexpression of PKN2 resulted in suppressed tumor growth compared to the control group (Figure 4A–C). The changes in the body weight of the mice during the model treatment are shown in Figure S2B. We further evaluated the effects of PKN2 overexpression on the survival and body weight of mice in the subcutaneous colon cancer model (Figure S2). The results demonstrated that, compared with the control group, PKN2 overexpression had a longer survival period and relatively minor impact on body weight. Confocal imaging revealed a significantly weaker CD31 signal in PKN2 overexpressing tumors, while a stronger CD31 signal was observed in control tumors, indicating a reduced MVD in PKN2 overexpressing tumors (Figure 4D–E). Furthermore, VEGFA and bFGF levels were examined in the tumor tissues, showing a significant downregulation of both factors in PKN2 overexpressing tumors (Figure 4F,G). These findings suggest that PKN2 inhibits colon growth and tumor angiogenesis and suppresses VEGFA and bFGF expression and secretion in vivo.

### 3.5 PKN2 Inhibited the Effect of HIF‐1α on the Expression of VEGFA and bFGF in Colon Cancer Cells

**FIGURE 5:**
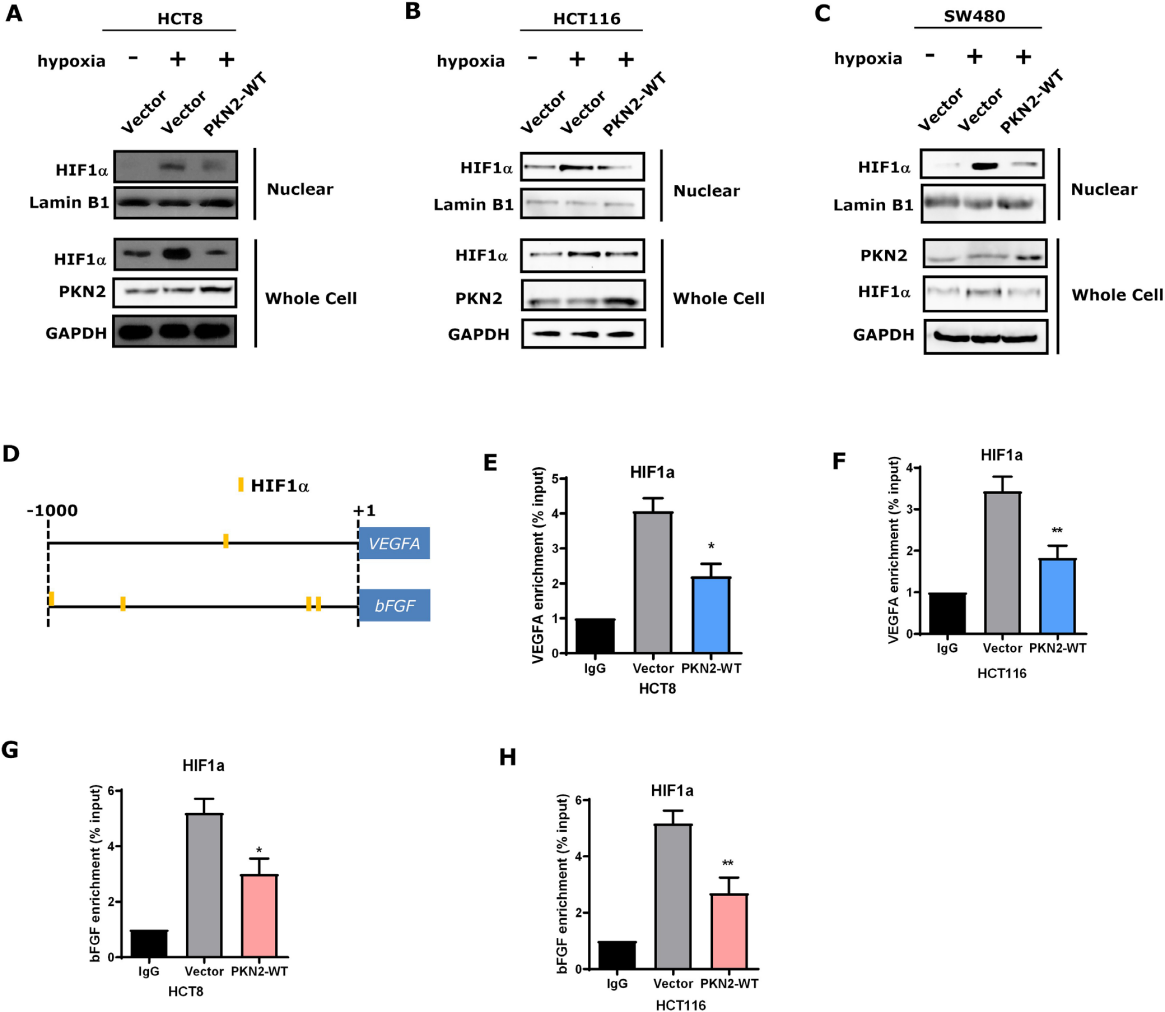
PKN2 suppressed the transcription of VEGFA and bFGF by inhibiting the binding of HIF‐1α to their promoters. (A–C) HCT8 cells (A), HCT116 cells (B), and SW480 cells (C) stably transfected with PKN2‐WT or control vector were cultured aerobically or anaerobically. HIF‐1α expression in whole cells and nuclei was evaluated by Western blot, using GAPDH and Lamin B1 as loading controls. (D) Schematic of predicted HIF‐1α binding sites on the −1000 to + 1 bp promoter regions of VEGFA and bFGF from JASPAR and TRANSFAC databases. (E–H) ChIP assays of HIF‐1α on the promoters of VEGFA and bFGF in HCT116 cells and HCT8 cells stably transduced with PKN2‐WT or control vector. **p < 0.01.

Our previously published work indicates that PKN2 inhibits the ERK signaling pathway in colon cancer cells. In this study, we aimed to identify other potential downstream signaling pathways regulated by PKN2. We analyzed our previous study's transcription factor microarray data [10] to explore PKN2‐regulated transcription factors in colon cancer cells. The ERK inhibitor U0126 was used to suppress the ERK pathway. As shown in Figure S3A,B, the relative transcriptional activity of HIF‐1α was significantly higher in PKN2‐knocked‐down HT‐29 cells. We further explored the effect of PKN2 on HIF‐1α in colon cancer cells under hypoxic conditions. Western blot analysis showed that hypoxia promotes the stability of HIF‐1α, while overexpression of PKN2 significantly downregulated the expression and nuclear internalization of HIF‐1α in colon cancer cells(Figure 5A–C). JASPAR and PROMO online databases were used to explore possible binding sites of the transcription factor HIF‐1α on VEGFA and bFGF gene promoter sequences. We identified several potential binding sites of HIF‐1α in the promoters of VEGFA and bFGF (−1000 to +1 kb) (Figure 5D). ChIP assays were performed to confirm the binding between HIF‐1α and the promoters of VEGFA and bFGF, as well as the effect of PKN2 on the binding. As shown in Figure 5E–H, HIF‐1α bound to the promoters of VEGFA and bFGF, while PKN2 overexpression inhibited HIF‐1α to DNA in both HCT8 and HCT116 cells. These data suggested that PKN2 suppressed the transcription of VEGFA and bFGF by inhibiting the binding of HIF‐1α to their promoters.

### 3.6 PKN2 Binds to HIF‐1 and Induces Phosphorylation‐ and Ubiquitination‐Mediated Degradation of HIF‐1α

**FIGURE 6:**
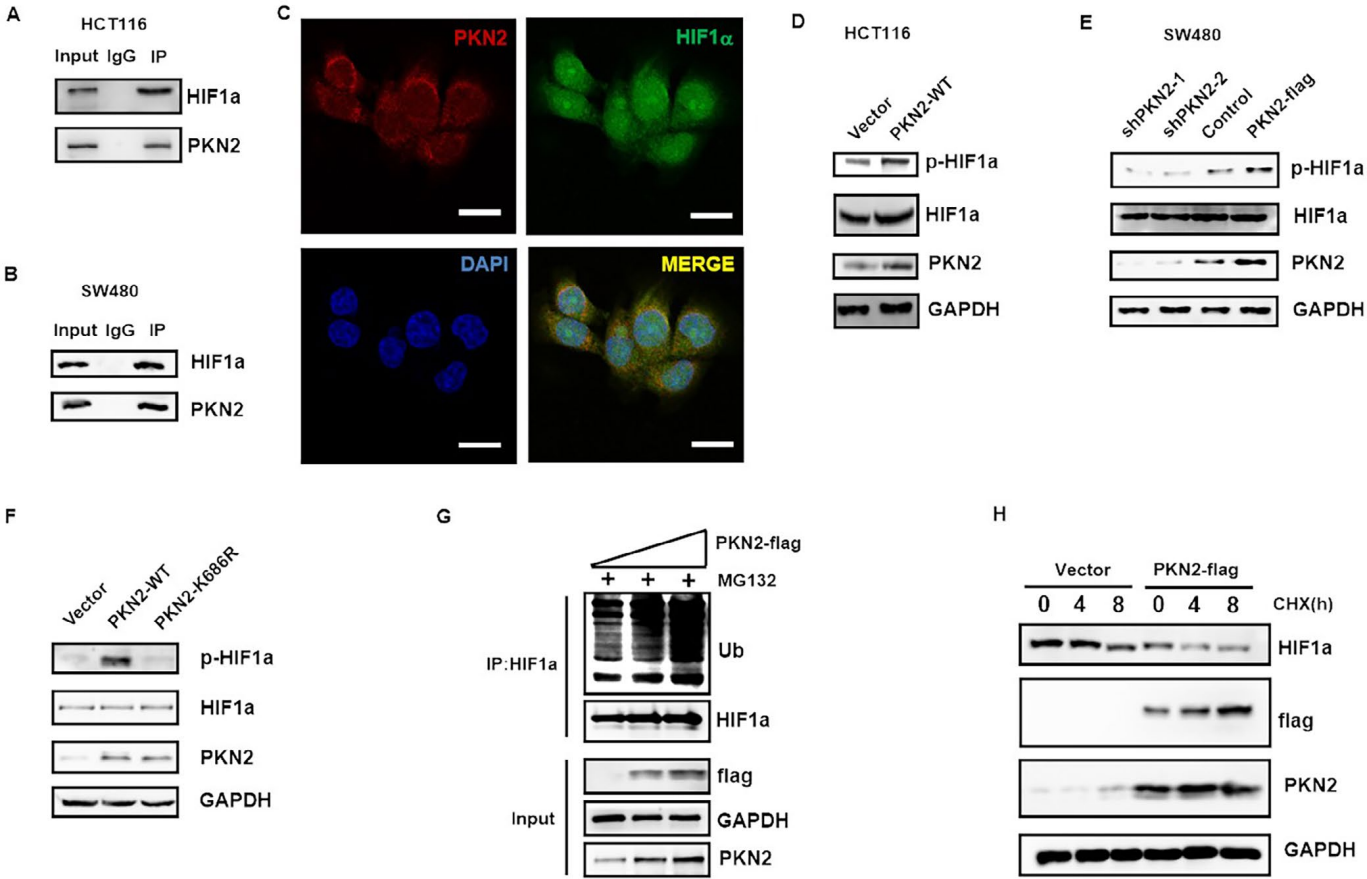
PKN2 interacts with HIF‐1α and promotes its ubiquitination and degradation. (A, B) HCT116 cell lysates were immunoprecipitated with anti‐ PKN2 antibody and coprecipitated HIF‐1α was detected by Western blot. (C) Confocal microscopy of HCT116 cells stained with anti‐PKN2 and anti‐HIF‐1α antibodies. (D) HCT116 cells that stably expressed PKN2‐flag or vector control were analyzed for HIF‐1α and pHIF‐1α expression by Western blot. (E) The expression of HIF‐1α and pHIF‐1α in control, PKN2 overexpression, and PKN2 knockdown SW480 cells were measured by Western blot. (F) HIF‐1α and pHIF‐1α expression in PKN2‐WT, PKN2‐K686R, and control HCT116 cells. (G) HCT116 cells stably expressing 3 μg or 6 μg PKN2‐flag or vector control were treated with 5 μM MG132 for 6 h. Cell lysates were immunoprecipitated with an anti‐HIF‐1α antibody and blotted for ubiquitin. (H) HCT116 cells transfected with indicated constructs and treated with 10 μg/mL CHX for the indicated time. The expression of HIF‐1α was detected by Western blotting, using GAPDH as an internal reference.

Next, we explored the regulatory effect of PKN2 on HIF‐1α. The coimmunoprecipitation and confocal studies showed that PKN2 directly binds to HIF‐1α in colon cancer cells (Figure 6A–C). Next, the HIF‐1α and phosphorylated HIF‐1α levels were measured. As shown in Figure 6D,E, the phosphorylation of HIF‐1α was increased in colon cancer cells with high PKN2 expression, while it was decreased in cells with PKN2 knockdown. However, overexpression of PKN2 K686R did not affect HIF‐1α phosphorylation (Figure 6F). These results suggest that PKN2 promotes the phosphorylation of HIF‐1α, with the K686 residue playing a key role.

Furthermore, the ubiquitination of HIF‐1α was detected in colon cancer cells transfected with low and high doses of PKN2 plasmid. The result showed that the ubiquitination of HIF‐1α increased in the PKN2 overexpression group, and the ubiquitination level of HIF‐1α increased with the PKN2 expression level (Figure 6G). This finding suggests that PKN2 can promote the ubiquitination of HIF‐1α. In addition, we used cycloheximide (CHX) to inhibit protein synthesis in the culture system. As shown in Figure 6H, the expression level of HIF‐1α in the PKN2 overexpression group became significantly lower with an increase in the duration of exposure to CHX. This finding suggests that PKN2 promotes the degradation of HIF‐1α. These results indicate that PKN2 directly binds to HIF‐1α and increases its phosphorylation, leading to the ubiquitination and degradation of HIF‐1α.

## 4 Discussion

The function of PKN2 in tumor angiogenesis has never been established. In the present study, we discovered that PKN2 expression correlated with tumor MVD in patients with colon cancer. Additionally, PKN2 inhibited angiogenesis induced by colon cancer cells in vitro under hypoxic and normoxic conditions. The inhibitory effect of PKN2 on angiogenesis was also confirmed in a mouse tumor model.

In our previous study, we found that PKN2 had no significant effect on the proliferation of colon cancer cells in vitro. However, it suppressed the M2 phenotype polarization of tumor‐associated macrophages in colon cancer. PKN2 expression was higher in normal colon tissue than in polyps, adenomas, and metastatic adenocarcinomas, decreasing gradually in these conditions. PKN2 expression was higher in the early stage of colon cancer [10]. Our findings also indicated that PKN2‐overexpressing colon cancer cell lines were enriched in MAPK and PI3K signaling pathways, and PKN2 inhibited the phosphorylation of Erk1/2. Erk1/2 activation also promotes the transcriptional activity of HIF‐1 and induces the phosphorylation and shift of HIF‐1α [13]. This study further explored whether PKN2 inhibits colon cancer growth through alternative pathways. We focused on the direct effect of PKN2 on key factors related to angiogenesis in colon cancer.

CD31 is commonly used as a vascular marker, while MVD indicates angiogenesis in tissues [14]. Tube formation by human endothelial cells is used as an in vitro marker of angiogenesis [15]. Our study demonstrated that PKN2 expression negatively correlates with both CD31 expression and MVD. Additionally, overexpressed PKN2 from colon cells induced decreased tube formation by EA.hy926 cells, suggesting that PKN2 plays a crucial role in angiogenesis.

VEGFA increases vascular permeability and promotes plasma protein and fibrin extravasation to the extracellular space and transformation into vascularized connective tissue [16]. A high VEGFA level increases the interstitial pressure, resulting in limited efficacy of traditional antitumor therapies. The overexpression of VEGFA is closely related to late tumor stage, high postoperative metastasis, and poor clinical outcomes in colorectal cancer [17]. bFGF stimulates the proliferation and migration of endothelial and tumor cells by binding to its receptor (FGFR), which is expressed on their cell surfaces [18, 19]. Colon cancer cells can express high levels of VEGFA and bFGF and promote angiogenesis through their paracrine effects [20]. Our study showed that PKN2 inhibited the expression and secretion of VEGFA and bFGF by colon cancer cells.

HIF‐1α is one of the major transcription factors that controls the expression of angiogenic genes, thereby promoting angiogenesis [21]. Early research suggests that hypoxic conditions induce HIF‐1α. Recent studies have shown that HIF‐1α is also overexpressed under nonanerobic conditions [22]. HIF‐1α is regarded as a key indicator of tumor neovascularization. The aberrant activation of HIF‐1α is mainly responsible for the overexpression of VEGFA and contributes to tumor progression [23]. It has been reported that HIF‐1α binds to the HRE in the promoter region of VEGFA [24]. Our study also revealed that HIF‐1α binds to the promoter of VEGFA. Our study showed for the first time that HIF‐1α binds to the promoter of bFGF. We also showed for the first time that PKN2 binds directly with HIF‐1α to regulate its activity. Furthermore, PKN2 suppressed the transcription of VEGFA and bFGF by inhibiting the binding of HIF‐1α to their promoter sequences.

HIF‐1α mRNA undergoes continuous translation into protein within cells. However, under normal oxygen tension, the level of HIF‐1α remains remarkably low due to persistent degradation by the ubiquitin‐proteasome system [25]. Suppressed HIF‐1α ubiquitination and degradation by DDR1 was reported to be involved in the malignant progression of gastric cancer [26]. In colorectal cancer, the transcriptional activity and stability of HIF‐1α were also reported to be regulated by 14‐3‐3σ [27]. Our findings demonstrate that the upregulation of PKN2 can enhance the phosphorylation of HIF‐1α and facilitate its ubiquitination and subsequent degradation, leading to a decrease in its level. These results suggest that posttranscriptional modifications may contribute to PKN2‐induced modulation of HIF‐1α expression. Moreover, the overexpression of PKN2 in the K686R mutant, which abolished the ATP binding and reduced the catalytic activity of the protein [28], significantly diminished the phosphorylation of HIF‐1α compared to wild‐type PKN2. This finding indicates that K686 may be a critical residue for PKN2‐mediated regulation of HIF‐1α.

Functional research on PKN2 in tumors is limited. Studies suggest that PKN2 may play a pivotal role in tumor growth, invasion, and metastasis. PKN2 appears to have a dual role in promoting or suppressing cancer, depending on the tumor type. PKN2 has been shown to contribute to motility pathways in prostate cancer cells and to facilitate gastric cancer metastasis [29]. Additionally, PKN2 is required for survival triple‐negative breast cancer cells [30]. However, our study suggests that PKN2 exerts a tumor suppressor effect in colon cancer. This can be attributed to the distinct tumor microenvironments across different tumors, such as regulation mediated by specific lncRNAs, microRNAs, and hormone signaling crosstalk. Furthermore, it may also involve modulation of PKN2 function through mutations or alterations in the expression levels of specific genes within various tumor types. Further investigation into the tissue‐specific functions of PKN2 may elucidate the dual role of PKN2 in cancer development across different tumor types. Inhibiting angiogenesis often exerts effective antitumor effects in highly vascularized tumors; however, blocking a single angiogenic pathway can induce hypoxia within the tumor microenvironment, thereby promoting more invasive phenotypes in tumor cells. Current research indicates that colon cancer tissues have an abundant blood supply, and anti‐VEGF treatment has proven effective. Considering the antiangiogenic role of PKN2 demonstrated in our study, enhancing the expression or activity of PKN2 may represent a promising therapeutic strategy for colon cancer. However, considering PKN2's diverse and significant physiological functions, the potential off‐target effects of PKN2 activation in normal tissues raise concerns regarding unintended consequences. Activating PKN2 may lead to cardiac dysfunction and heart failure [31], induce pulmonary fibrosis [32] impair actin cytoskeleton assembly and alterecell adhesion. Until now, no selective activators of PKN2 have been reported. It is crucial to investigate therapeutic approaches that can selectively activate PKN2 in tumor cells using nanoparticle‐based delivery systems. However, the development of PKN2 activators or targeted delivery systems presents several potential challenges, including ensuring safety, achieving biocompatibility, minimizing immunogenicity, enhancing targeting efficiency and precision, improving cellular uptake, and clarifying metabolic pathways [33]. We propose that PEGylation might be incorporated during the development of these activators to mitigate immunogenicity, reduce in vivo clearance rates, and prolong the drug's half‐life.

There are some limitations to our study. First, our study did not explore whether the PKN2 expression level correlates with treatment response to antiangiogenic therapies due to the limited number of samples from patients who underwent these therapies. Subsequently, we will conduct a more in‐depth analysis of patient samples obtained from clinical trials involving antiangiogenic agents. Further clinical studies are required to determine whether PKN2 can serve as a prognostic biomarker or identify patients who might benefit from antiangiogenic therapies. Additionally, the interaction between PKN2 and other signaling pathways, such as PI3K/AKT and Wnt/β‐catenin, needs further exploration.

In conclusion, our data indicated for the first time that PKN2 inhibits tumor angiogenesis in colon cancer. Our study also proposed a novel mechanism by which PKN2 regulates the dynamic equilibrium of HIF‐1α expression. We demonstrated that PKN2 promotes the ubiquitination and degradation of HIF‐1α in colon cancer cells via direct binding to HIF‐1α. Moreover, PKN2 inhibited the expression and secretion of VEGFA and bFGF by colon cancer cells by inhibiting the binding of HIF‐1α to the VEGFA and bFGF promoter regions.

## Conflicts of Interest

The authors declare no conflicts of interest.

## Supporting information


Data S1.

## Data Availability

The data that support the findings of this study are available on request from the corresponding author. The data are not publicly available due to privacy or ethical restrictions.
