# Supplementary material for: PKN2 Inhibits VEGFA and bFGF‐Mediated Angiogenesis by Targeting HIF‐1α in Colon Cancer
Source: Kaohsiung J Med Sci. 2025 Jun 14;41(7):e70050. doi: 10.1002/kjm2.70050 (PMC12412600; doi:10.1002/kjm2.70050)
Supplement: Supplementary file 1 — Data S1. [file KJM2-41-e70050-s001.docx]

**Supplementary Table S1. Sequence of primers for qRT-PCR.**

| **Gene** | **Forward primer** | **Reverse primer** |
| --- | --- | --- |
| ***VEGFA (human)*** | **5′- -3′** ACGTACTTGCAGATCTCTCACC | **5′- -3′** ATGGGCTGCTTCTTCCAACA |
| ***VEGFB (human)*** | **5′- -3′**TGACATCACCCATCCCACTCC | **5′- -3′**GGGCAGGCAGTCTGTATTGA |
| ***VEGFC(human)*** | **5′- -3′**CCCGCCTCTCCAAAAAGCTA | **5′- -3′**TGGACACAGACCGTAACTGC |
| ***VEGFD(human)*** | **5′- -3′**GAACACCAGCACCTCGTACA | **5′- -3′**ACAGACACACTCGCAACGAT |
| ***PIGF(human)*** | **5′- -3′**GGGAAAGACCATGGCAGATGA | **5′- -3′**GCCATACCTTGCACTACGGTT |
| ***SCDGF(human)*** | **5′- -3′**TGCCTCTTGTTTCCAATGCCT | **5′- -3′**AAGGGTGCTTGCCTGAAGAG |
| ***aFGF(human)*** | **5′- -3′**CTGCAGTAGCCTGGAGGTTC | **5′- -3′**GAGGGCTGTACCTCTCTCCA |
| ***bFGF (human)*** | **5′- -3′**TCCACCTATAATTGGTCAAAGTGGT | **5′- -3′**CATCAGTTACCAGCTCCCCC |
| ***FGF5(human)*** | **5′- -3′**TACCCAGGGAGACCGGAAAT | **5′- -3′**CTGAAGGAGTGTGCTCCCTG |
| ***FGF6 (human)*** | **5′- -3′**GCGTGGTGAGTCTCTTTGGA | **5′- -3′**CCGCTTTACCCGTCCGTATT |
| ***Int-2(human)*** | **5′- -3′** GGGGACGACTCTATGCTTCG | **5′- -3′** CCGTTCACAGACACGTACCA |
| ***Hst/ks3(human)*** | **5′- -3′** ATACCCGGTGCAGGAGTTTG | **5′- -3′** CCGACGCTATCTGCAATCCT |
| ***Ang-1(human)*** | **5′- -3′** TGCTGAACGGTCACACAGAG | **5′- -3′** GTACTGCCAGCACACTCCTT |
| ***Ang-2 (human)*** | **5′- -3′** GGGCATAATTGTGCTTGACTGG | **5′- -3′** CCGTTCGAACTGTCTCACCA |
| ***Ang-3 (human)*** | **5′- -3′** TCAACAGACACAAAAAGACATCAT | **5′- -3′** CCAGATCAGGTGGTGGCATT |
| ***Ang-4 (human)*** | **5′- -3′** ACCAGCTATACAGGGTGGTG | **5′- -3′** CCCACCATTGGTCATGGGAA |
|  | | |

**Supplementary Table S2.  Sequence of primers for ChIP.**

| **Gene** | **Forward primer** | **Reverse primer** |
| --- | --- | --- |
| ***VEGFA for HIF1α*** | **5′- -3′ AGATCTGTGTGTCCCTCTCC** | **5′- -3′ GTGAGGTTACGTGCGGACA** |
| ***bFGF for HIF1α*** | **5′--3′ TGCATCTGGAGTTAAAGCCTTC** | **5′--3′ TGCAAGTATGGACGTGCCTC** |
|  | | |

**Supplementary Figure S1.**

**
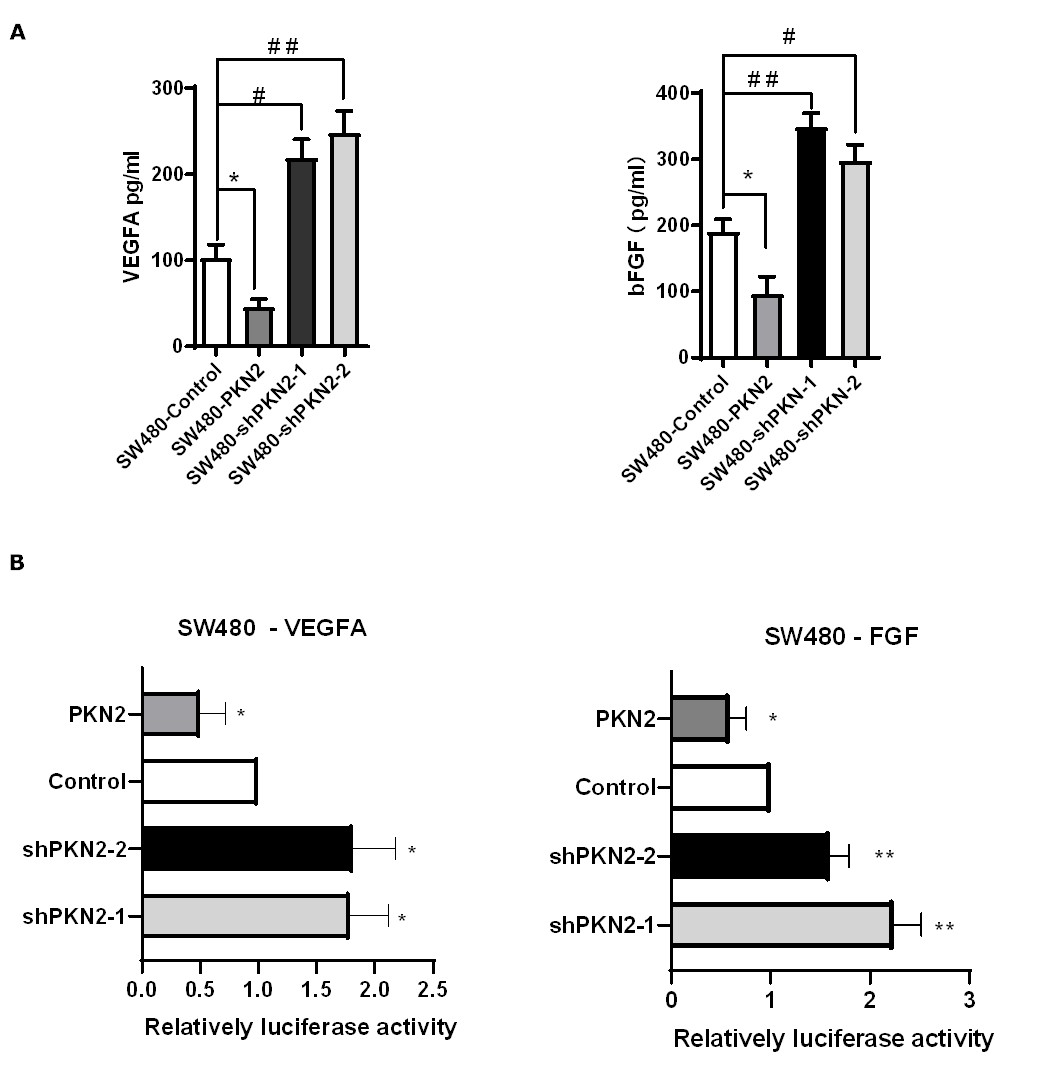
**

A. The levels of VEGFA and bFGF in the cell culture supernatant were determined using ELISA for control/ PKN2 overexpression/ PKN2 knockdown SW480 cells. B. The transcription factor binding activities of VEGFA and bFGF in control/ PKN2 overexpression/ PKN2 knockdown SW480 cells. Relative fold-change in luciferase activity is shown.**, P < 0.01; ***, P < 0.001 versus Control.

**Supplementary Figure S2.**

**
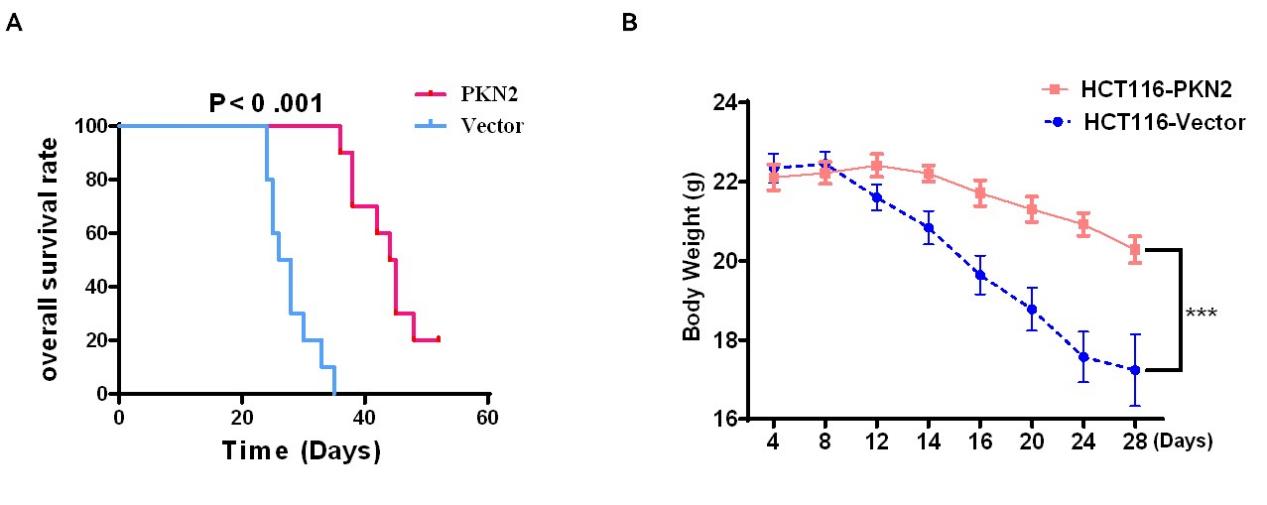
**

1. Overall Survival time of nude mice with subcutaneous injection of PKN2 overexpressed/control HCT116 cells（n=10）. B. Body weight of mice of the two groups. ***p<0.001.

**
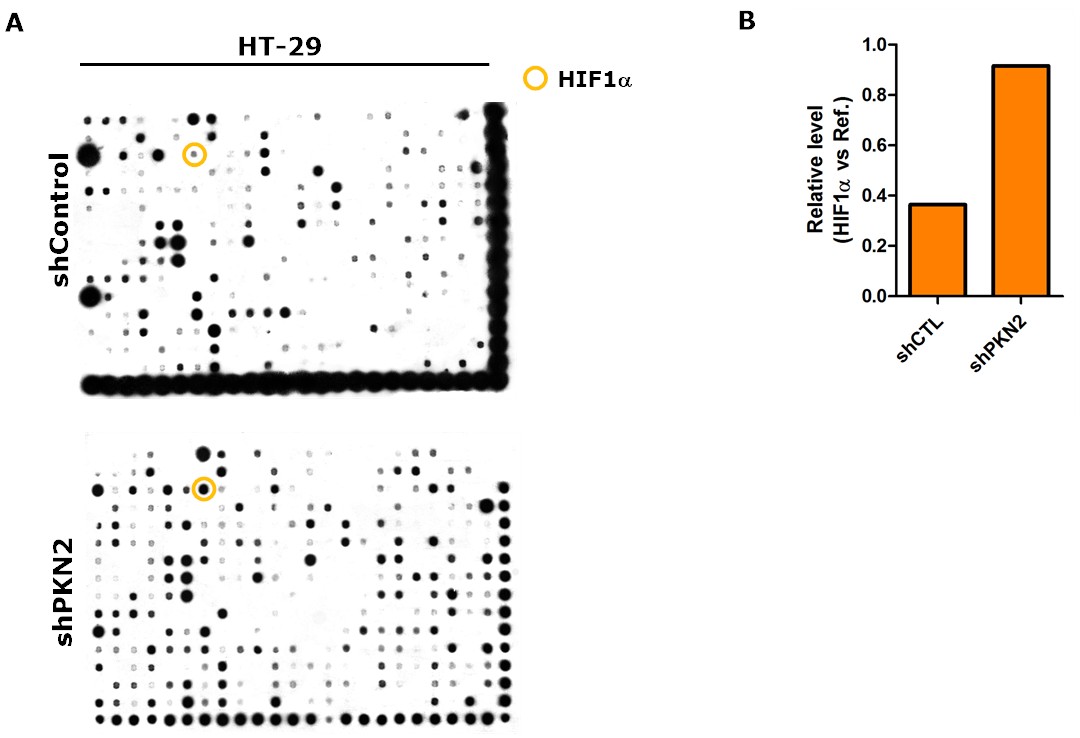
**

**Supplementary Figure S3.**

A. HT-29 cells were stably transduced with shPKN2 or control shRNA and treated with U0126 (1μM) for 24 hours. Transcriptional factor activity arrays were performed. B Relative transcriptional activity levels of HIF1α was indicated.
